# A membrane computing simulator of trans-hierarchical antibiotic resistance evolution dynamics in nested ecological compartments (ARES)

**DOI:** 10.1186/s13062-015-0070-9

**Published:** 2015-08-05

**Authors:** Marcelino Campos, Carlos Llorens, José M. Sempere, Ricardo Futami, Irene Rodriguez, Purificación Carrasco, Rafael Capilla, Amparo Latorre, Teresa M. Coque, Andres Moya, Fernando Baquero

**Affiliations:** Department of Microbiology, Ramón y Cajal University Hospital, IRYCIS, Carretera de Colmenar Viejo, km. 9,100, 28034 Madrid, Spain; Biotechvana, Valencia, CEEI Building, Benjamin Franklin Av. 12, Valencia Technological Park, 46980 Paterna, Spain; Department of Information Systems and Computation (DSIC), Polytechnic University of Valencia, Camino de Vera, 46022 Valencia, Spain; Antibiotic Resistance and Bacterial Virulence Unit (HRYC-CSIC), associated to the Superior Council of Scientific Investigations (CSIC), Madrid, Spain; Network Research Center for Epidemiology and Public Health (CIBER-ESP), Madrid, Spain; Cavanilles Institute of Biodiversity and Evolutionary Biology, University of Valencia, C/ Catedrático José Beltrán 2, 46980 Paterna, Valencia Spain; Foundation for the Promotion of Health and Biomedical Research in the Valencian Community (FISABIO) - Public Health, Avenida de Cataluña 21, 46020 Valencia, Spain

**Keywords:** Membrane computing, P-system, Antibiotic resistance, Essential nesting

## Abstract

**Background:**

Antibiotic resistance is a major biomedical problem upon which public health systems demand solutions to construe the dynamics and epidemiological risk of resistant bacteria in anthropogenically-altered environments. The implementation of computable models with reciprocity within and between levels of biological organization (i.e. essential nesting) is central for studying antibiotic resistances. Antibiotic resistance is not just the result of antibiotic-driven selection but more properly the consequence of a complex hierarchy of processes shaping the ecology and evolution of the distinct subcellular, cellular and supra-cellular vehicles involved in the dissemination of resistance genes. Such a complex background motivated us to explore the P-system standards of membrane computing an innovative natural computing formalism that abstracts the notion of movement across membranes to simulate antibiotic resistance evolution processes across nested levels of micro- and macro-environmental organization in a given ecosystem.

**Results:**

In this article, we introduce ARES (Antibiotic Resistance Evolution Simulator) a software device that simulates P-system model scenarios with five types of nested computing membranes oriented to emulate a hierarchy of eco-biological compartments, i.e. a) peripheral ecosystem; b) local environment; c) reservoir of supplies; d) animal host; and e) host’s associated bacterial organisms (microbiome). Computational objects emulating molecular entities such as plasmids, antibiotic resistance genes, antimicrobials, and/or other substances can be introduced into this framework and may interact and evolve together with the membranes, according to a set of pre-established rules and specifications. ARES has been implemented as an online server and offers additional tools for storage and model editing and downstream analysis.

**Conclusions:**

The stochastic nature of the P-system model implemented in ARES explicitly links within and between host dynamics into a simulation, with feedback reciprocity among the different units of selection influenced by antibiotic exposure at various ecological levels. ARES offers the possibility of modeling predictive multilevel scenarios of antibiotic resistance evolution that can be interrogated, edited and re-simulated if necessary, with different parameters, until a correct model description of the process in the real world is convincingly approached. ARES can be accessed at http://gydb.org/ares.

**Reviewers:**

This article was reviewed by Eugene V. Koonin, and Eric Bapteste.

## Background

Antibiotic resistance (AR) is a serious biomedical problem upon which public health systems urge for new R&D strategies to prevent the emergence and dissemination of resistant bacteria in human inhabited environments [1–6]. The discovery and clinical use of antimicrobials during the past 20th century has changed the course of medicine and the human lifestyle by reducing mortality from bacterial infections. However, the use or misuse of antibiotics, medicines and drugs may lead bacteria to become tolerant to the action of antimicrobials, eventually making standard treatments ineffective and thus challenging key medical practices as intensive care medicine, transplantation, or the therapy of immuno-compromised patients [2, 5, 7]. AR is also a subject of particular interest to food-chain stakeholders who use antimicrobials as growth-promoting additives until recently or for preventing cross-infection in food production areas [7–12]. Under antibiotic exposure, farms have become significant reservoirs for antibiotic resistant microorganisms, which in turn can be transmitted from food-producing animals and plants to humans [7–13]. The matter is to understand the risks and the unintended consequences of the anthropogenic use and release of antimicrobial agents into the biosphere, which without appropriate measures might drive the world towards a post-antibiotic era where mortality due to untreatable and fatal infections may become customary, particularly in underdeveloped areas of the world [3–5, 12, 14]. Understanding AR evolution is however a complex issue, since it is not just the result of antibiotic-driven selection of mutant resistant bacterial clones as it is frequently considered, but more properly the consequence of a variety of trans-hierarchical interactions between all biological vehicles involved in the dissemination of the genetic information involving AR [15–18]. We are talking about the following biological entities: genetic platforms, transposons and/or plasmids (here called subcellular replicators), bacterial cells, genetic exchange communities (GEC, communities in which the interchange of genetic material occurs frequently), microbiota, host individuals and host communities. In other words, not only AR genes can be carriers of AR genetic information, but also all other biological units in which AR genes can be successively located at different subcellular, cellular and supra-cellular nested levels of the ecosystem [19–21]. It is important to note the need of understanding the effect of the whole nested frames associated to AR to establish a comprehensive “parameter space” able to describe the multidimensional evolution of antibiotic resistance (Table 1, for more details see [22]). All the aforesaid carriers are units of selection that can be simultaneously and independently chosen at different environmental levels including “invironmental” or microbiotic ecosystems [23]. Therefore, we should expect a high complexity in between-hosts demographical dynamics, as the host colonized or infected by resistant bacteria usually belongs to a population of interacting individuals that in turn belong to a community of interacting bacterial species. Note, for instance, that lice and keds of pets and farm animals (*Melophagus ovinus, Linognathus vituli, Heterodoxus spiniger*) or pest-insects such as the house fly (*Musca domestica*) and the German cockroach *Blattella germanica*, which are known to play a significant role as reservoirs and vectors of opportunistic bacterial pathogens that are often resistant to antibiotics [24–26] as they move, freely and indiscriminately, from filth and animal waste to food, allowing resistant bacteria to explore new habitats in hospitals, community settings and food facilities [27–29]. This type of complex scenarios, where bacterial resistant populations and their genetic platforms containing AR genes move because they are selected, and evolve because of their promiscuous migration, establish the precondition to visualize AR not only as a matter of biological function, but also as information flow processing. For these reasons, AR evolution is without doubt one of the major biomedical challenges for researchers in epidemiology and systems biology.

**Table 1:** Key-nested frames associated to AR: a complex parameter space

a) Density of colonized and colonizable hosts with antibiotic resistant bacteria
b) Population sizes of bacteria per host during colonization and infection
c) Susceptibility to colonization of hosts, including age, gender, ethnicity, nutrition, illness-facilitated colonization
d) Frequency of between-hosts interactions i.e. ,human-to-human or animal-human interactions
e) Host natural and acquired immune response to colonizing organisms
f) Ecological parameters of colonizable areas, including interaction with local microbiota and frequency and type of antibiotic-resistant commensals
g) Migration and dispersal
h) Antibiotic and biocide exposure and overall density of antibiotic use, type of antibiotics and mode of action, dosage and duration of therapy, adherence to therapy, selective antibiotic concentrations, antibiotic combinations
i) Mode of transmission of resistant organisms from the environment to hosts
j) Transmission rates between hosts (antibiotic treated and not-treated, infected, and not-infected)
k) Time of contact between hosts
l) Hygiene, infection control, sanitation
m) Food, and drinking water contamination by resistant bacteria and host exposure
n) Environmental contamination by resistant organisms, including soil, sewage and water

Interestingly, from the epistemological interaction of system biology, computer science and mathematics, a variety of models have arisen in the last decades to connect performances at different scales. These models are known as nested or embedded models (for a review see [30]) and have been used to acceptably address specific questions involving within-host dynamics enclosed in a model of between-host epidemiological scenarios. Nested models are classified as “inessential” when the within-host dynamic influences between-host processes but not *vice versa*, or “essential” when there is a reciprocal feedback between levels of organization. In particular, AR modeling requires an essential nested model; any alteration of the carriers in any specific resistance trait, or in their mechanisms of variation and mobilization (mutation, recombination, transposition, horizontal gene transfer, migration) may influence the dynamics of other units of higher and lower hierarchy, having logical consequences on the frequency and dissemination of AR genes and, therefore, evolutionary and/or ecological consequences on bacterial population [31, 32]. Until not too long ago, the difficulty to model this type of scenarios with essential nesting was an important limitation to feasibly study AR evolution processes. However, exciting new opportunities have recently arisen from a natural computing formalism inspired on the structure and functioning of biological cells, called membrane computing [33–35]. Membrane computing conceives any biological system as a hierarchical construct where the flow of materials can be interpreted as computing processes. In particular, membrane computing offers a versatile framework known as P-system that consists of a hierarchical membrane structure of nested compartments where multisets of objects are located and can move across membranes evolving according to a finite number of given rules. Membrane computing have been proved to be universal models of computation [34] and has been successfully used to model oscillatory systems [36], processes of signal transduction [37, 38], gene regulation control [39], quorum sensing [40], meta-populations [41] and ecosystems [42, 43] thus suggesting that anything that can be computed can be done so as a P-system. For more details on the different approaches reached under membrane computing, see [44], or refer to the official websites of the membrane computing community [45] and P–Lingua [46] the programming language used for the development of P-systems.

In this paper we introduce a new P-system model designed for computing at three levels of organization (subcellular, cellular, supra-cellular) through the software implementation of a simulator we call Antibiotic Resistance Evolution Simulator (ARES). The general aim of ARES is to facilitate predictive computational models on the potential trans-hierarchical response of AR to particular interventions in specific scenarios. The simulator´s project is a work in progress, requiring constant refinements derived from the experiences (“experiments”) of costumers. The first version here introduced, is a prototype that offers a predefined layout composed of five types of nested-membranes that conceptually emulate an ecosystem hierarchy of biological boundaries based on population environmental areas, reservoirs, host populations and bacterial lineages of opportunistic pathogens. Granted to the implementation a friendly-to-use front-end interface, the user is allowed to define a starting configuration of elements (subcellular vehicles, antimicrobials and other substances) inhabiting the aforesaid membranes, specifications and rules according to which both elements and membranes evolve through a number of iterations. ARES is hosted at the GyDB Project [47] a database for research of mobile genetic elements (relevant carriers in the study of AR), and has been launched as an online server accessible at http://gydb.org/ares.

## Methods

### P-system model for simulating ecosystems with nested ecological boundaries

AR is a process of multilevel selection of nested units where the distinct resistance-carriers (gene vehicles) influence each other for selection and introgressive crossing of resistance to antibiotics at different environmental levels (subcellular, cellular and supracellular) [18, 20, 48]. This is an eco-biological model that can be formally generalized according to the following tuple:

$$ {\displaystyle \prod =\left(V,\kern0.5em \mu, \kern0.5em {w}_1,\kern0.5em {w}_2,\dots, {w}_n,\left({R}_1,{\rho}_1\right), \dots \left({R}_n,{\rho}_n\right)\right)} $$

where *V* is a working alphabet of objects; *μ* is a membrane structure consisting of *n* membranes labeled 1, 2, . . . , *n* represents a rooted tree; and *w*_1_, . . . , *w*_n_ are strings over *V* that represent multisets of objects initially placed in the structure of *n* membranes, which, from that point on will be referred as ecological boundaries (EBs) through the rest of this article.

In the *V* alphabet, our model takes into account the following entities to be treated as objects:

- Bacterial resistance genes computed either as an independent unit or constituting a combination together with a particular subcellular replicator (for instance a plamid). Here, we use the set of symbols *AR*_*i*_ to describe genes encoding AR, where *i* denotes the object’s identity. Should an AR gene is designated in its single form (AR-like) it will be considered as a genomic gene (i.e. present as a locus within the bacterial host genome) by the model, but if it is attached to a particular subcellular replicator then it will be considered to be part of the subcellular replicator (i.e. carried by the subcellular replicator).
- Subcellular replicators inside bacterial cells such as plasmids, integrative-conjugative elements (ICE), transposons, or any other genetic element with self-replication ability. For the sake of simplicity, in this first version we only consider plasmid-like objects, which our model computes with the set of symbols *PL*_*i*_. As previously indicated above, the model permits simulation of plasmids carrying AR genes by introducing a regular expression that define complex objects as follows: let us to consider *k* different plasmids and *j* different AR genes, then a complex object for the combination of plasmids with genes belongs to the following expression (with λ being an empty string in the absence of a name): (PL_i_ + … + PL_k_)(AR_i_+λ)… (AR_j_+λ). For example the strings “PL_1_”, “PL_1_-AR_1_”, “PL_1_-AR_2_”, and “PL_1_-AR_1_-AR_2_” respectively correspond to objects representing four different forms of the same plasmid – “not carrying AR genes”, “carrying gene AR_1_”, “carrying gene AR_2_” and “carrying both AR genes”. For computational sake, the current version of our P-system model permits only simulation of plasmids carrying up to two different AR genes.
- External chemicals and/or biomaterials released into the environment, including any kind of molecule inhibiting bacterial growth such as antibiotics and biocides. We use the set of symbols *A*_*i*_ to describe these objects.
- Management Clocks are objects labeled with *G*_*i*_ symbols used to periodically add objects to specific membranes according to ecosystem influences expected to be cyclic.

In the membrane structure *μ* the model implements five hierarchical EBs labeled as *ECO*, *P*_*i*_*, RS*_*i*_, *H*_*i*_ and *B*_*i*_

- *ECO* is the skin EB representing the peripheral ecosystem or ultimate container for all populations and environments.
- *P*_*i*_ is the second EB type in the membrane framework level that designates simulation of particular environmental areas of the ecosystem for the spread of hosts and resistant bacteria and eventually other bacterial pathogens (inhabitable spaces, areas for food acquisition, and other eventual infrastructures).
- *H*_*i*_ is a type of EB on the third level of nesting within the membrane framework hierarchy. We use *H-*like EBs to define host individuals carrying microbiota (host-specific assemblies of bacterial microorganisms, defined by its microbiota composition).
- *RS*_*i*_ is another type of EB, also on the third framework level, that the model uses in order to abstract (when suitable) the computation of three different types of physical or conceptual reservoirs. Two of these are called Food and Water supplies and are used during the simulation as reservoir-EBs of these resources. It is worth to note that for computational sake food and water are not treated as objects but as quantitative internal resources of the P-system being necessary for hosts´ live that must therefore be periodically generated by management clocks and consumed by the simulated hosts according to the rates stated by the users in the configuration of rules. Once the host population growth outpaces the availability of food and water the model activates an internal malthusian rule that randomly kills (eliminates from the simulation) a number of hosts equivalent to the population surplus. The third RS-like EB is called “Sewage” and it refers to any body of water conveying all water-carried waste (either natural or anthropogenic) being removed from a community. Sewage can also be used to simulate the stool remains (or fecal droppings) periodically released by H-like individuals to the environment. The three RS-like types of EBs are represented only once within each (P-like) environment and although they are providers of water and food they may also contain substances and microbial contamination released through the droppings of animal hosts and the conversion of dead animals into food. In other words, the use of reservoirs allows the user to simulate supplies of food and water but also recycling of microbiota released into the ecosystem by animal hosts (H-like membranes) during the final act of digestion or to turn into food any organism that dies or is predated by other organisms.
- *B*_*ij*_^±^ is the last EB level of the hierarchy contemplated in our model and it is used to simulate bacterial cells. Each cell has several attributes here defined as follows: the superscript “plus/minus” is used to indicate if the cell is gram-negative or gram positive; the subscript *i* is used to represent cell populations as lineages term here used to highlight that the user can design the simulation of a microbiome according to a common historical offspring of cells at any taxonomical level (a lineage can therefore refer to a domain, phylum, class, order, family, genus, species and clones, depending on the experiment); *j* is used to assign two or more cells to a particular or unique community of cells or GEC. For instance, within a microbiome, the user can define a subset of cells to belong to GEC_1_ and another subset to belong to GEC_2_ using the subscript differentiation.

The dynamics, specificity and behavior of the distinct membranes and objects during the P-system simulation are administrated by a finite set of rules (*R*) fixed to each membrane that can be ranked by order of priorities (*ρ*). Particularly, our P-system model considers rules for the processes of transition, interactions and social behavior, birth, death, inactivation, and evolution of the distinct objects and membranes. The model also considers rules called specifications for ecosystem resource limitations in space and time (as limits in space, or life expectancy). Following are some examples of rules described in the formal definition followed by the membrane computing community, and where for the sake of simplicity, we omit priorities and stochastic parameters.

Example 1: transition rules with movement to hierarchically adjacent regions:

“Substance Ai enters bacterial cells” or “Substance Ai leaves bacterial cells”.

$$ \Big[{u}_i\left[\kern0.5em \right]{}_{j\kern0.5em }\Big]_k\to {\left[\kern0.15em {\left[{u}_i\mathit{\hbox{'}}\right]}_j\right]}_k\kern0.5em (inside) $$

$$ {\left[{\left[{u}_i\right]}_j\right]}_k\to {\left[{u}_i\mathit{\hbox{'}}\kern0.15em {\left[\kern0.5em \right]}_j\right]}_k\kern0.5em (outside) $$

Example 2: transition rule with movement to twin adjacent regions:

“A bacterial cell transmits a plasmid to other members of the same GEC”.

$$ {\left[{u}_i\right]}_j\ {\left[\kern0.5em \right]}_i\to {\left[\kern0.5em \right]}_j\kern0.5em {\left[{u}_i\mathit{\hbox{'}}\right]}_i $$

Example 3: active membrane rules with movement to twin adjacent regions:

“An individual host transmits bacteria to another individual”.

$$ {\left[{\left[\right]}_k\right]}_j\kern0.5em {\left[\kern0.5em \right]}_j\to {\left[\kern0.5em \right]}_j{\left[\kern0.5em {\left[\kern0.5em \right]}_k\right]}_i $$

Here, region *k* is moved from region *j* to region *i,* that is at the same level. Then, regions inside region *k* can be moved according to their corresponding rules.

Example 4: active membrane rules for membrane division:

“Growth of bacterial cells j in region i.”

$$ {\left[{\left[\right]}_j\kern0.5em \right]}_i\to {\left[\ {\left[\right]}_j\ {\left[\ \right]}_j\ \right]}_i $$

Here, the content of region *j* is copied together with its rules and all the membranes it contains in a hierarchical manner.

**Fig. 1:**
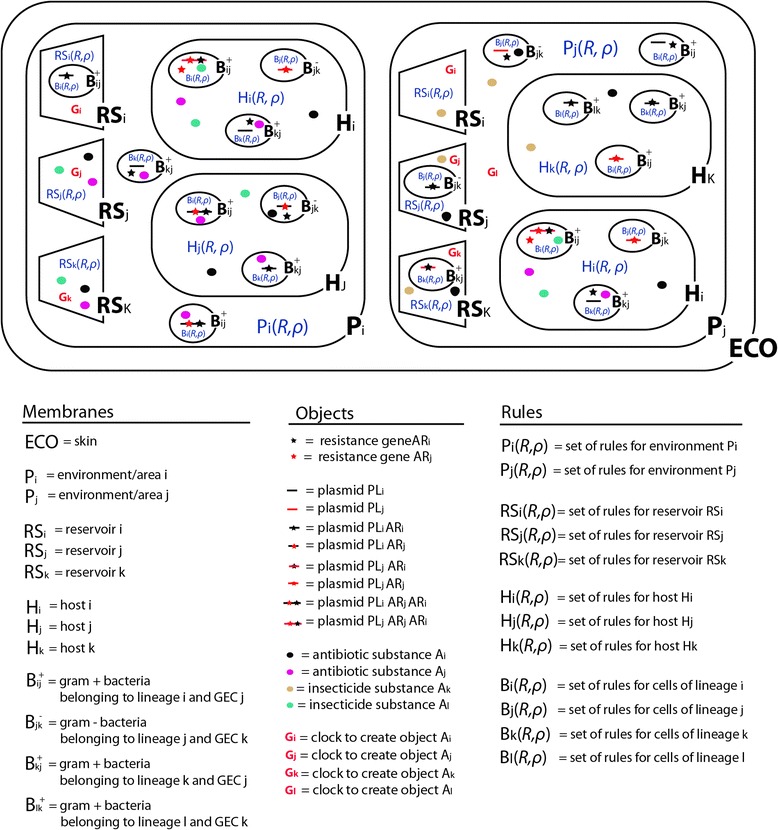
P-system model for AR evolution in complex ecosystems. Venn diagram representation showing of the framework of membranes and vocabulary of objects, on which our P-system model is based; membranes are illustrated as nested diagrams labeled at bottom according to the model´s code of symbols we use for referring membranes; objects are also represented using symbols summarized below the figure; and rules assigned to each membrane area are, for simplicity´s sake, indicated as text indications colored green

In summary, our P-system consists of a membrane structure composed of five types of EBs and a working alphabet *V* of objects whose interactive feedback is determined by the set of rules assigned at every EB. This framework can be graphically represented as a Venn diagram (Fig. 1). As shown in the figure, the container diagram (ECO) represents the skin EB. Within ECO, the two next diagrams designated as – P_i_ and P_j_ – represent two P-like environmental EBs (the user can however design as many P-like EBs as required). P-like EBs are allowed to contain RS-like, H-like and B-like EBs (represented as diagrams of smaller size). RS-like EBs (designated as *i, j, k*) represent food, water and sewage reservoirs and are allowed to contain B-like membranes (but not H-like membranes). H-like EBs can be distinguished in subtypes (social classes, species, etc.) using subscript assignations. For example, in the figure we contemplate 3 populations (*i, j, k*) that may be respectively composed of a number of individuals (for instance 100, 50 and 150, etc.). Each H-like EB is allowed to contain a number of internal B-like EBs (but not RS-like EBs) defining its intrinsic microbiota. B-like EBs can be placed not only within RS-like and H-like EBs but also in P-like EBs and can be differentiated in lineages to which gram and GEC status can be assigned using sub- and superscripts. The status of Gram positive or Gram-negative organisms is assigned using a superscript with two states (minus and plus). In the figure we observe four subtypes (*i*_*_*_*, j, k, l*) according to the left subscript. Those labeled with the left subscript *j* belong the GEC-j and those labeled with the subscript *k* belong to GEC-k. Those having the superscript plus are considered to be gram-positive cells, and those assigned the superscript minus are gram-negative. Logically, population size can also be assigned to each lineage (for example 10^9^ cells per bacterial lineage). The working alphabet is composed of four types of objects (also differentiated in subtypes) summarized below the Venn diagram. In particular the figure shows two AR-like objects (*i, j*) defining two different AR genes; four A-like objects (*i, j, k, l*) defining four distinct substances (for instance two antibiotics and two insecticides with different properties); eight PL-like objects representing two plasmids (*i, j*) each one with four possible states (without AR genes, carrying an AR_i_ gene, an AR_j_ gene or carrying both AR genes); and four G-like objects (*i, j, k, l*) representing management clocks. AR-like and PL-like objects are restricted to B-like EBs but they can move from a B-like EB into another (to emulate horizontal transfer events). Note, however, that AR objects can be only transferred when carried by a plasmid object. A-like and G-like objects are allowed in all EBs (excepting ECO which is the ultimate container) as they either define substances expected to spread across all environments or periodical actions (in the case of G-like management clocks) stated by the user. Finally, every EB is assigned a set of specific rules (R) designed and tuned with the aim to govern the dynamic of interactions and evolutionary events within each EB according to given priorities (*ρ*), parameters and conditions indicated by the user.

## Results and discussion

### Introducing ARES: simulator device core and server implementation

The P-system model previously defined in Methods was implemented as a simulator software, which was programmed using the Java object-oriented computer programming language [49] and P-lingua foundations [50]. In particular, the simulator reads an xml file that contains the starting configuration of a P-system and run simulation of the P-system case study configured during a user-defined number of interactions contemplating four steps per iteration; 1) *step of evolution* where all rules for evolution and interaction are applied; 2) *step of movement* where all rules for movement apply; 3) *step of growth* where all membranes allowed to divide do so. After each step, an updating pre-step prepares the model for the next one. The rules applying to each region act simultaneously, in a parallel way, to all the objects and regions, and the stochastic behavior of the system is achieved by applying the rules according to their stochastic parameters in a naive probabilistic manner. Rules apply if: a) the objects in need of the rule application are in the region and b) there is no rule with higher priority that uses common objects. The transition from one configuration to the next is carried out by applying all the rules at every region in a non-deterministic maximal parallel mode; the system is always running from one configuration to the next and only halts if no rule can be applied. Hence, the halting configuration contains the output of the system and the final configuration. In addition, stochastic parameters to model the population dynamics of the system are also introduced in the rules.

The simulator has been installed within an engine core within a 4× 6 Core Server with Linux OS and 128 GB of RAM, and has been coupled with the following sub-systems; 1) a MySQL Management System for storage of P -system configurations; 2) an engine for output conversion to CSV format; 3) the output´s archive, which is an repository of output folders; 4) a server section to upload training tutorials; 5) a collection of scripts for statistical analysis developed using the R programming language [51]; 6) A front-end interface layout to manage all other sub-systems, programmed in a PHP framework on Laravel 4 following the Model–View–Controller pattern an architectural model where server interfaces are interchangeable [52]. This Infrastructure is what we call ARES.

### Managing ARES

**Fig. 2:**
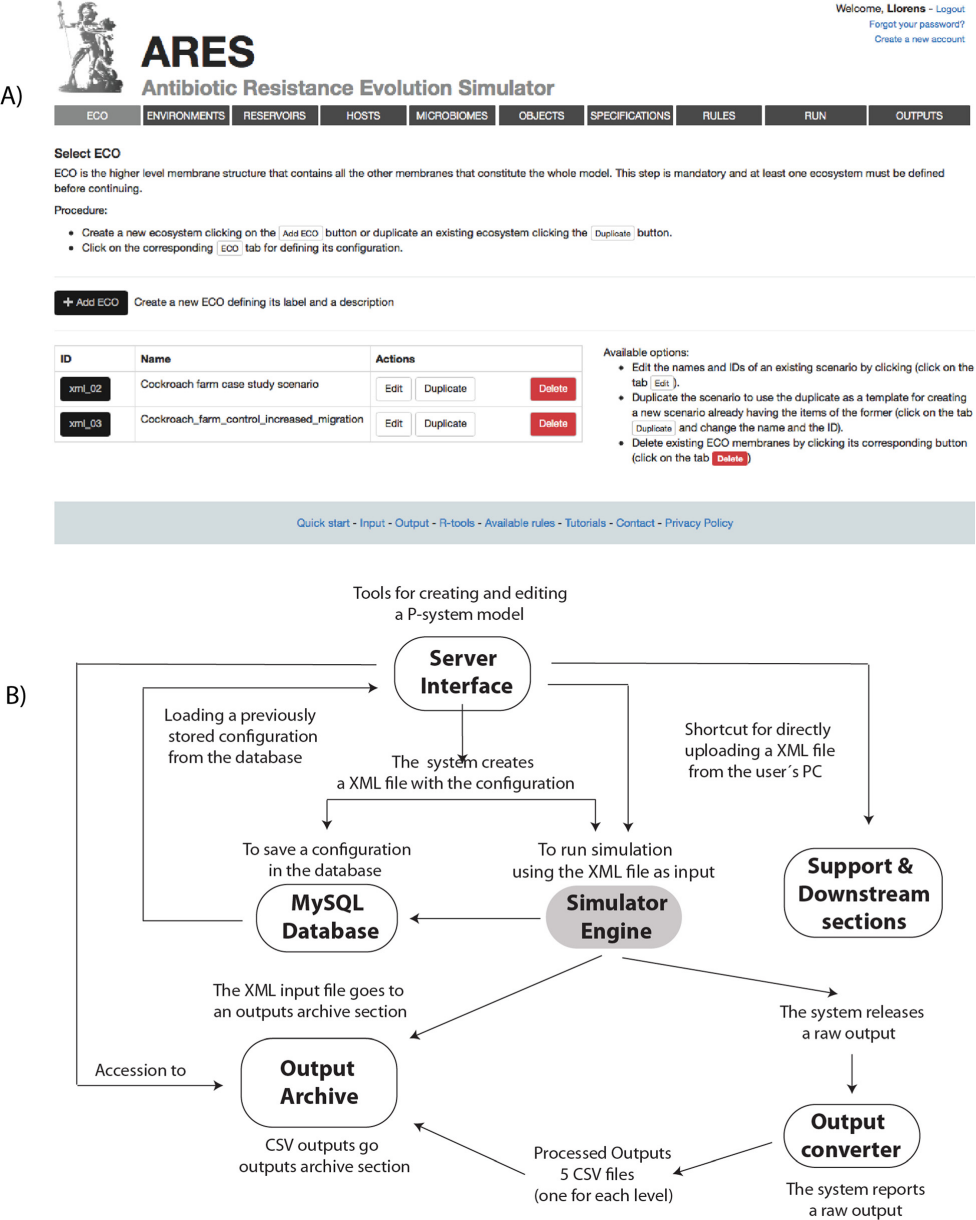
ARES interface and server organization. **a** Screenshot of the ARES interface. The interface implements a menu that gives access to the distinct server forms that apply for configuration, storage and simulation of P-system model scenarios. At the bottom of the interface the user can access other support sections for managing ARES of for statistical interrogation of the output generated by the simulator device. **b** ARES sever scheme and workflow for creation, edition and simulation of P-system model scenarios

Users can simulate dynamics of AR evolution using ARES to design a P-system model scenario adapted to the case study specified by the user and then run simulation of this scenario as many times as necessary correcting parameters until a realistic description of the AR process is approximated or validated if real world observations are available. Use of ARES is free, but its accession is password protected in order to allow the users to open and maintain a user account that will needed to store and run model projects in private session (a simulation may last hours or even days depending on the complexity of a P-system scenario). ARES is managed via an easy-to-use interface that implements a centralized menu (Fig. 2a) for accessing the system of forms the user need to sequentially complete in order to introduce the starting configuration of a P-system scenario, run a simulation, and access the results. All menu-forms accessible with this menu can be navigated back and forth for editing the P-system configuration, change or add EBs, objects and rules where or when necessary. A scheme of the whole ARES infrastructure and the workflow for configuration and simulation of P-system scenarios is depicted on Fig. 2b. The usual procedure can be synthetized in the following steps. The form designated as “*ECO”* must be first accessed (via menu) and completed to create the P basal skin EB; then *“ENVIRONMENTS”* has to be accessed to configure as many P-like (environmental) EBs as needed within ECO; next, *“RESERVOIRS*” and *“HOSTS*” must be filled to configure RS-like (reservoirs) and H-like (hosts) EBs within the previously created P EBs; after this, “*MICROBIOMES”* must be used to configure a series of B-like (bacterial) EBs that can be either placed within the previously created P-, RS- and H-like EBs; then *“OBJECTS”* must be used to create as many as PL- (plasmids), AR- (AR genes), A-like (antibiotics and/or other substances), and G-like (clocks) objects as required within the previously created EBs (except for ECO, since it is the skin membrane); finally, the forms *“SPECIFICATIONS”* and *“RULES”* must be completed to state the rules assigned to each EB by selecting them from a list of pre-designed rules provided in an understandable and generalized way allowing the user to choose and tune rules with the values and the parameters needed to approximate the frequencies, behaviors, conditions and priorities that govern the dynamic of interactions among the different membranes and objects of the P-system model to be simulated.

Once the P-system starting configuration has been defined, it is automatically written to an xml file (which is the input into the simulator), which is stored in ARES. The form *“RUN”* is an interface that provides accession to the simulator engine core accompanied by a list of all xml files ready for simulation. The user only needs to select an xml file from such a list, then determine the number of iterations the simulation will last (each iteration is set to be correspond to one day) and run the simulation. It is worth to note that xml files can be exported or imported from the user’s PC as convenience, using the *“RUN”* interface*.*

Once the simulation starts, ARES automatically creates a folder (labeled with the name given to the P-system during the configuration) in the outputs’ archive assigned to this particular simulation and then generates the xml input file that is placed within this ouput folder. When the simulation finishes the system first delivers a raw output, which is a plain file containing the sampling counts per iteration of all simulated objects and EBs. The raw output is difficult to manage because of the default format it encloses. To overcome this difficulty, ARES implements an output converter engine that processes and splits the raw output into a set of 5 formatted csv files appointed as *ECO-like.csv, P-like.csv, RS-like.csv, H-like.csv, B-like.csv.* These files constitute together the output that ARES delivers after simulation for the user into the output’s archive (the output’s archive can be accessed using the “*OUTPUTS*” tab in the ARES menu). Each csv contains the counts for each EB and object sampled at the P-system EB level referred in the file name. For instance, *ECO-like.csv* has the counts of all EB and objects sampled at the ecosystem level, *H-like.csv* has the counts of all objects and EBs sampled in the H-like EBs but not in those of higher levels (i.e. ECO and P-like); and *B-like.csv* file has the counts of all objects sampled in B-like EBs. In each csv, iterations correspond with the rows and are organized in ascending order (being the starting configurations the first count in the file) and membranes and objects correspond with the columns.

ARES also offers other sections for user support, which can be accessed via the submenu available at the footer of the ARES interface. 3 of these sections called “*R-TOOLS”,* “*TUTORIALS”,* and *“AVAILABLE RULES”* are of particular interest*.* The first section (*R-TOOLS*) gives access to an interface (also managed via menu) offering the users different scripts mainly but not exclusively developed in “R”, for downstream interrogation of csv outputs. Note however that the use of *R-TOOLS* is not a mandatory task as csvs are open plain files that can be processed using any other tool or statistical packages such as Excel, Gnuplot, Matlab and Mathematica, etc. The second section (“*TUTORIALS”*) is a repository where users can upload and download tutorials for P-system configuration and management of ARES (see also the section below, “Tutorials and training material”). Finally the third section (“*AVAILABLE RULES”*) is a section where we summarize all pre-designed rules to date available for each type of EB in order to let the user to make preliminary evaluations of the rules to take for a particular simulation before creating the starting configuration of the P-system. This section also includes a form for users to make if necessary any specific suggestion for the implementation of new rules not yet available.

### Tutorials and training material

Although management of ARES is quite intuitive, the design and preparation of the starting configuration of a P-system model scenario can be an arduous task for researchers not familiarized with membrane computing (configuration of items, assignation of rules, etc.). Taking this into primary consideration, we have prepared two tutorials aimed to give the reader some training material that can be downloaded from the *“TUTORIALS”* section of ARES under the labels “Nosocomial Scenario” and “Two cockroach farms” respectively. The first tutorial contemplates a simplified nosocomial scenario provided with the sole objective of allowing the user to take the first steps in learning how to configure an exemplary P-system creating two membrane environments (a community and a hospital environment), host EBs (for instance, patients), microbial communities within hosts, composed of distinct bacterial EBs and plasmids, carrying AR genes objects within the bacterial EBs. The tutorial also exemplifies how to create clocks to introduce other objects such as antibiotics in the simulation or how to configure and tune a basic package of rules. The second tutorial contemplates a more elaborated scenario that can be addressed after completing the first tutorial. This tutorial specifically focuses on the simulation of two populations of *B. germanica* (a model insect organism able to implant an intestinal microbiota similar to that of humans [53–56]) respectively emplaced in two separate cages with the possibility of migrating from one to the other. These two boxes conceptually represent environmental EBs for hospital and urban-community individuals, here designated as P_1_ and P_2_. Cockroaches of both farms are hosts (H-like EBs) carrying the same intestinal microbiota, which according to Carrasco et al. [56] is predominantly composed of eight bacterial lineages (B-like EBs). Four of these cellular lineages will be simulated as Gram-negative, while the four other will be Gram-positive. All bacterial cells of all lineages are allowed to carry three distinct types of intracellular plasmids (PL_1_, PL_2_ and PL_3_) capable of horizontal transfer. One of these (PL_1_) is carrier of an AR gene (AR_1_) conferring resistance to a gram-negative specific antibiotic designated as “A_1_”while another plasmid type (PL_2_) carries an AR gene (AR_2_) offering resistance to a gram-positive specific antibiotic labeled as A_2_. In the starting configuration, the third plasmid type (PL_3_) does not carry AR genes but during the course of the simulation it is allowed to recruit any of two types of AR genes simulared. Each P-environment has food and water supplies (RS-like membranes) to simulate the feeding of H individuals. The overall aim of this tutorial is to compare the response of the microbial communities in two main scenarios (control and case study) simulated under three distinct interventions (increase of the rate of migration, increase of antibiotic dosage, and fumigation). In total, the tutorial considers eight P-system scenarios (designated from xml_1_ to xml_8_) and provides indications about how to prepare, configure and simulate the aforesaid scenarios during 600 iterations (equivalent to 600 days). Although the second tutorial is provided solely for demonstration purposes, it is worth noting that the ecological scenario of this tutorial is in advanced process of being implemented in the real world, such a way providing a powerful tool for “experimental epidemiology” of antibiotic resistance. Such an epidemiological model will be useful for the validation of ARES, confronting predictions with real outcomes when the changes are introduced in the computing model and in the experimental “two cockroach farms” setting.

## Conclusions

ARES is a new membrane computing simulator we have launched with the aim to help researchers develop computational models oriented to help elucidation of hidden aspects of the epidemiological and ecological complex patterns of AR that cannot be easily traced in the real world, due to both practical and complexity reasons. The underlying computational model of ARES is a P-system that differs from other models (including other previously published P-systems) in that both the framework and set of rules permit the user to simulate stochastic dynamics at different environmental (subcellular, cellular and supracellular) levels of the simulated ecosystem. This ability is what allows the user to asses the reciprocal feedback between the different carriers involved in the dissemination of AR genes, edit the configuration of the model scenario and then re-run the simulation with changing the parameters until a correct description of the AR process is approximated according to real world observations. ARES is a project in continuous progress and, therefore, a prototype in which we are working for future implementations and improvement, including an experimental epidemiology model (the “two cockroach farms model”). The project is open to all other experts interested in contributing expertise and criticisms.

## Reviewers´ comments and response

### Reviewer´s report 1: Eugene Koonin

Reviewer´s comment:

I must indicate that I am not an expert in membrane computing. That said, my impression is that ARES is a highly promising, flexible platform for modeling the complex dynamics of antibiotic resistance evolution. The description of the model is quite logical and meticulous. Given the obvious, overarching importance of the study of antibiotic resistance, I expect that this tool will be in high demand in the research community.

Authors´ response: *Thank you very much for your positive comments and feedback. We hope the device and the formalism to be of interest for researchers working in predictive models for AR evolution or in System Biology. It is important to also recognize all the previously work done by the membrane computing community whose know-how (cited in the “Background” of this article), has been an important starting reference for us in order to conceptualize and design the P-system model we introduce in this article.*

### Reviewer’s report 2: Erik Bapteste

Reviewer´s comment:

This work is stimulating to read and remarkable by its ambition: simulating complex systems and following the evolutionary dynamics of a diversity of objects within these systems. This manuscript introduces a rather intuitive formalization for these two tasks, taking advantage of P-systems. However intuitive these approaches are, I fell that an additional illustration, typically a Venn diagram for a simulation considered worthy of interest by the authors, would greatly help most readers to go beyond some of the rather abstract (and potentially discouraging) formalism used in the main text, e.g. when P-systems are described by pseudo-equations. Such a Venn diagram would also probably help to immediately appreciate what hierarchically adjacent regions? or twin adjacent regions? are on a concrete example.

Authors´ response: *Thank you very much for your positive evaluation and criticisms. This second version of the manuscript includes a Venn diagram (designated as Fig.* *1**) where the framework of membranes and objects contemplated by the model are represented (and discussed in text).*

Reviewer´s comment:

When I tested ARES on line, the website was operational, and user friendly. The proposed implemented specifications seem sound and numerous enough to be of use to interested scientists, or to make them feel scared by the many decisions one must make to fully benefit from this approach. Typically, not being an expert on the evolution of such systems, it was difficult for me to appreciate what sets of parameters and what rules were realistic ones. While I realize that in principle comparing simulations results with the biological reality may help one to a posteriori decipher what parameters were indeed realistic, I am a bit skeptical that in practice this strategy will necessarily work so well for several reasons:

- How can one measure the similarity between simulated results and the biological reality? It might be useful to implement such comparative measures in ARES.
- How can one know when the simulation results are significantly close to the reality to be approximated? Some statistics would be needed here.
- Consistently, how can one discriminate between multiple scenarios producing comparable results what scenario is more realistic, if any, especially with so many parameters? Is there a way to compare, say, the complexity of two models with equally likely outputs? I guess these might become tasks for the future, should ARES evolve in a way that helps its users to explore parameters ranges in a statistically meaningful framework.

Authors´ response: *Conceptually speaking, membrane computing is easy and intuitive but it is true that designing a P-system and preparing the xml input file for running the P system with an appropriate starting configuration is a daunting task because of the reasons you indicate. This motivated us to design ARES as friendly as possible in order to let the users to deal with membrane computing without being an expert in membrane computing. However, it is also true that to appropriately manage ARES, the user must do an first effort in getting familiar with at least the basic principles of membrane computing and also make another effort in getting some training. These issues, motivated us to create several support sections in ARES (see also our response to the minor comments). One of these sections (that called R-TOOLS) consists of a collection of R-scripts managed via interface for statistical interrogation of the ARES outputs. Evidently, ARES, and of course the aforemenctioned R-TOOLS section, are both work in progress with obvious limitations but the project is scalable. In this first release we have designed the basal P-system model and have programmed the software implementation of this P-system. At present, we are preparing new implementations. The 3 questions you address are excellent examples of new improvements we take note in order to implement them as soon as possible. Meanwhile, it is worth noting that the output released by ARES is a plain file the user is free to process with any third-party statistical package and statistical model or test (ANOVA, MANOVA, etc.) for comparing/discriminating the outputs of one or more scenarios among them or with empirical observations, which in turn can help the user to tune the starting configuration and re-run a simulation as many time as needed until a particular configuration approximates the real world observation.*

Reviewer´s minor comment 1:

- Some generic terms related to P-systems, such as membrane structure? or regions?, could be misleading in this particular context of medicine and microbial evolution, and may deserve to be adjusted.

Authors´ response: *In this version we have used the term “Ecological Boundary” (EB) when referring to particular membranes of the P-system framework. Hope you will find this term more appropiate.*

Reviewer´s minor comment 2:

- It is easy to get lost in the numerous options (specifications and such): maybe having examples of what are considered as realistic parameters in some known environment (i.e. having default values associated with particular environments), or the possibility to run a simple pre-implemented case study might help the user to perform meaningful analyses ? Maybe such a pre-implemented P-system is just what the import environments? option already offers, but this option did not seem to work for me on line.

Authors´ response: *We have created a section called “TUTORIALS” within ARES that permits users to upload and share new tutorials with other users and where we also provide 2 tutorials (one very simple and the other a bit more complex) with material and indications allowing the user to get some training before starting with his/own P-systems. In addition we have also created a contact section for users support as well as another section for frequently asked questions (FAQs). Also, and considering your feedback, we have created another section called “AVAILABLE RULES” where all pre-designed rules to date implemented in ARES are listed with the aim to let the users pre-study the whole set of rules and then evaluate which rules are appropriate or not for their interests. This new section also includes a form for users to make us recommendations in regards of new rules not yet contemplated that we will also try to program as soon as possible. Finally, let us to make one clarification; at present, there are not pre-implemented P-systems in ARES but the possibility to re-use the complete (or partial) configuration of a P-system previously introduced and stored in ARES by the user. The option did not work for you because you do not have any P-system configuration in ARES previously stored. We have clarified this in FAQS and where correspond in the system of forms of ARES but we also take note in any case of this interesting suggestion - have a collection of P-system configuration modules (i.e. pieces) pre-implemented in ARES – for further improvements.*

Reviewer´s minor comment 3:

- For GEC descriptions, please explain what distinct numerical values will mean (i.e. will the same number mean that bacteria belong to the same gene exchange community, or will the value? 0? mean that bacteria do not belong to any GEC? Or does it indicate that a particular set of bacteria can be split into 3 GEC, when 3 is the value chosen?)

Authors´ response: *Done in text.*

Reviewer´s minor comment 4:

- The password on ARES must contain numbers, but it does not say this right away.

Authors´ response: *Amended.*

Reviewer´s minor comment 5:

- It might be nice to also have a Venn diagram as the output to compare the overall picture before?, and after simulation?

Authors´ response: *Originally we aimed to implement Venn diagrams in the way you suggest (at the beggining or at the end of a simulation) but we dismissed the idea because it is only viable when plotting small P-systems. Bear in mind that one expect to find distinct types and subtypes of EBs and objects in the starting (or final) configuration of a more or less regular P-system for AR evolution, as well as a variety of rules assigned to each EB subtype. Note that although the population size of some membranes and objects to plot can be defined in single units (no more than a ten), the population size of just one bacterial lineage could reach thousands or even millions of EBs. We are however, working in order to find a satisfactory graphical solution when representing P-system complex scenarios via ARES.*

## Abbreviations

AR: Antibiotic resistance
ARES: Antibiotic Resistance Evolution Simulator
EB: Ecological boundary
GEC: Genetic community
ICE: Integrative-conjugative elements
MVC: Model–view–controller
